# Surveillance of infectious-disease outbreaks on cruise ships: a fragmented system without a global registry

**DOI:** 10.3325/cmj.2026.67.322

**Published:** 2026-08

**Authors:** Aristomenis Exadaktylos, Aebhric O`Kelly, Isabella Demartini, Tyson Welzel

**Affiliations:** 1 University of Cyprus School of Medicine, Nicosia, Cyprus; 2 Faculty of Offshore & Maritime Medicine, The College of Remote and Offshore Medicine Foundation, Ghaxaq, Malta; *docaris@protonmail.com*; 3 Acuvera Advisory, Altendorf, Switzerland

Clinicians and public-health practitioners who seek a definitive record of infectious-disease outbreaks on cruise ships are often surprised that none exists. What is in place is a set of jurisdiction-specific systems operating under the International Health Regulations (2005), as amended in 2014, 2022, and 2024 (1). The Regulations require States Parties to maintain capacities to detect, assess, notify, and respond to public-health events and provide for health documents on arrival, including the Ship Sanitation Certificate and the Maritime Declaration of Health. The amended Regulations entered into force on September 19, 2025 (1). They do not, however, establish a consolidated global outbreak database. Whether a given event is recorded, and whether that record can be retrieved, therefore depends substantially on the port a vessel enters. This creates a structural distinction between an international legal framework for notification and preparedness and the much more fragmented systems through which shipboard events are actually recorded.

The largest openly searchable record is operated by the United States Centers for Disease Control and Prevention. Its Vessel Sanitation Program monitors acute gastroenteritis on cruise ships carrying 13 or more passengers that arrive at a US port within 15 days of a foreign call, using the Maritime Illness Database and Reporting System; ships submit standardized case reports before arrival, and a voyage is posted publicly once a defined proportion of those on board report gastrointestinal symptoms (2,3). The program provides an unusually transparent and useful source of outbreak information, including voyage-level reporting that can be searched retrospectively. Its remit is nevertheless the binding constraint: vessels that do not call at a US port and pathogens other than those causing gastroenteritis fall outside the openly published record (2). Thus, the US system should be viewed as a strong national surveillance model rather than a proxy for global cruise-ship disease surveillance.

Outside the United States, most outbreak data are held within national competent authorities rather than exposed as a searchable public record. European surveillance has been supported by common guidance and the Maritime Declaration of Health, but reporting requirements differ markedly. In a survey of competent authorities across European countries, only about 30% required the Declaration from all arriving ships, and most respondents reported gaps in surveillance and control (4). Such variation matters because cruise itineraries routinely cross several jurisdictions during a single voyage. An event may therefore be assessed by one port health authority, managed operationally by another, and become visible to a national surveillance system only if a specific reporting threshold is met. The absence of a common public data layer makes it difficult to estimate incidence, compare outbreaks between countries, or determine whether apparently isolated events represent recurring patterns.

A further limitation is the range of diseases the system was built to capture. The mature machinery centers on gastrointestinal illness, while broader surveillance has had to evolve in response to emerging threats (5). Disease-specific networks can nevertheless capture single pathogens well: the European Legionnaires’ Disease Surveillance Network systematically identifies travel- and cruise-associated cases. However, microbiological confirmation is low, with clinical isolates obtained for only about 6% of travel-associated cases in one annual data set, meaning that many clusters would not have been detected by national surveillance alone (6). The remaining gaps include onward transmission to shore, incomplete comparability between studies and surveillance systems, and limited coverage of zoonotic or environmentally driven events (5). These limitations suggest that future surveillance should not simply expand the number of diseases recorded but should also capture the epidemiological links between the vessel, passengers and crew, ports of call, and subsequent community transmission.

Importantly, Europe does have regional infrastructure that partly addresses this fragmentation. EU HEALTHY GATEWAYS, building on the EU SHIPSAN ACT initiatives, supports coordinated preparedness and passenger-ship inspections using the European Manual for Hygiene Standards and Communicable Disease Surveillance on Passenger Ships (7). The current European Manual brings together hygiene inspection standards, communicable-disease surveillance procedures, and guidance for prevention and management of disease events on passenger ships (7). The associated infrastructure also supports the recording of ship inspections and Ship Sanitation Certificates and facilitates information exchange on public-health events. These mechanisms are particularly relevant because they create common operational standards across participating European port authorities and can reduce duplication of inspections (7). They provide a practical basis for cross-border collaboration and standardization, but they remain regional surveillance and preparedness infrastructure rather than a comprehensive outbreak registry covering all cruise ships and all pathogens.

Cruise-ship outbreak surveillance is therefore patchwork rather than a register. For the clinician or investigator facing a possible shipboard outbreak, the practical course is to combine openly published sources with the relevant national authority, regional port-health infrastructure and surveillance network specific to the pathogen in question, rather than expecting a single point of reference. For policy, the more consequential point is that detection should not depend on a vessel’s itinerary. The priorities are harmonized case definitions and comparable reporting instruments, interoperable data-sharing between port states, and extension of surveillance beyond gastrointestinal and respiratory illness to zoonotic and environmental hazards. A useful next step would be a federated approach in which national and regional systems retain responsibility for their own data while sharing a minimum common data set. Such an approach could improve comparability without requiring the creation of a single central global registry.
